# Functional magnetic resonance imaging research in China

**DOI:** 10.1111/cns.13725

**Published:** 2021-09-07

**Authors:** Hongzan Sun, Yong He, Heqi Cao

**Affiliations:** ^1^ Department of Radiology Shengjing Hospital of China Medical University Shenyang China; ^2^ State Key Laboratory of Cognitive Neuroscience and Learning Beijing Normal University Beijing China; ^3^ Beijing Key Laboratory of Brain Imaging and Connectomics Beijing Normal University Beijing China; ^4^ IDG/McGovern Institute for Brain Research Beijing Normal University Beijing China; ^5^ Chinese Institute for Brain Research Beijing China; ^6^ Department of Health Sciences National Natural Science Foundation of China Beijing China

**Keywords:** brain connectivity, brain disorders, cognition, connectome, fMRI

## Abstract

Functional magnetic resonance imaging (fMRI) non‐invasively measures the activity of the human brain and provides a unique technological tool for investigating aspects of the human brain including cognition, development, and disorders. As one of the main funding agencies for basic research in China, the National Natural Scientific Foundation of China (NSFC) has initiated various research programs during the last two decades that are related to fMRI research. In this review, we collected and analyzed the metadata of the projects and published studies in research fields using fMRI that were funded by the NSFC. We observed a trend of increasing funding amounts from the NSFC for fMRI research, typically from the General Program and Key Program. Leading research institutes from economically developed municipalities and provinces received the most support and formed close collaboration relationships. Finally, we reviewed several representative achievements from research institutions in china, involving data analysis methods, brain connectomes, and computational platforms in addition to their applications in brain disorders.

## Graphical abstract

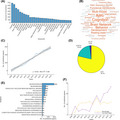

## 1 INTRODUCTION

Functional magnetic resonance imaging (fMRI) based on blood oxygen level‐dependent (BOLD) contrast offers a meritorious technique that can noninvasively measure the activity of the human brain in vivo.^1^ Traditional fMRI studies using a psychological task paradigm and activation detection have identified brain regions that relate to various functions from basic perception to high‐order cognition. On the other hand, Biswal et al.^2^ first described the existence of synchronization between signals of sensorimotor regions by using fMRI data obtained during the resting‐state, hailing a new start in the investigation of the intrinsic functional architecture of the human brain. After more than two decades of development, fMRI research has gradually formed a complete system with perspectives from focal activities and functional connections to complex networks and dynamic models. These methods have been widely used to investigate cognition, normal development and aging, and brain diseases, playing an indispensable role in brain science research. In China, increasing attention has been given to fMRI research, and such progress is continuously supported by the National Natural Science Foundation of China (NSFC). In this review, we seek to provide a glimpse into the landscape of funding and output from the NSFC in fMRI research.

### 1.1 Funding for fMRI research from the NSFC

**FIGURE 1:**
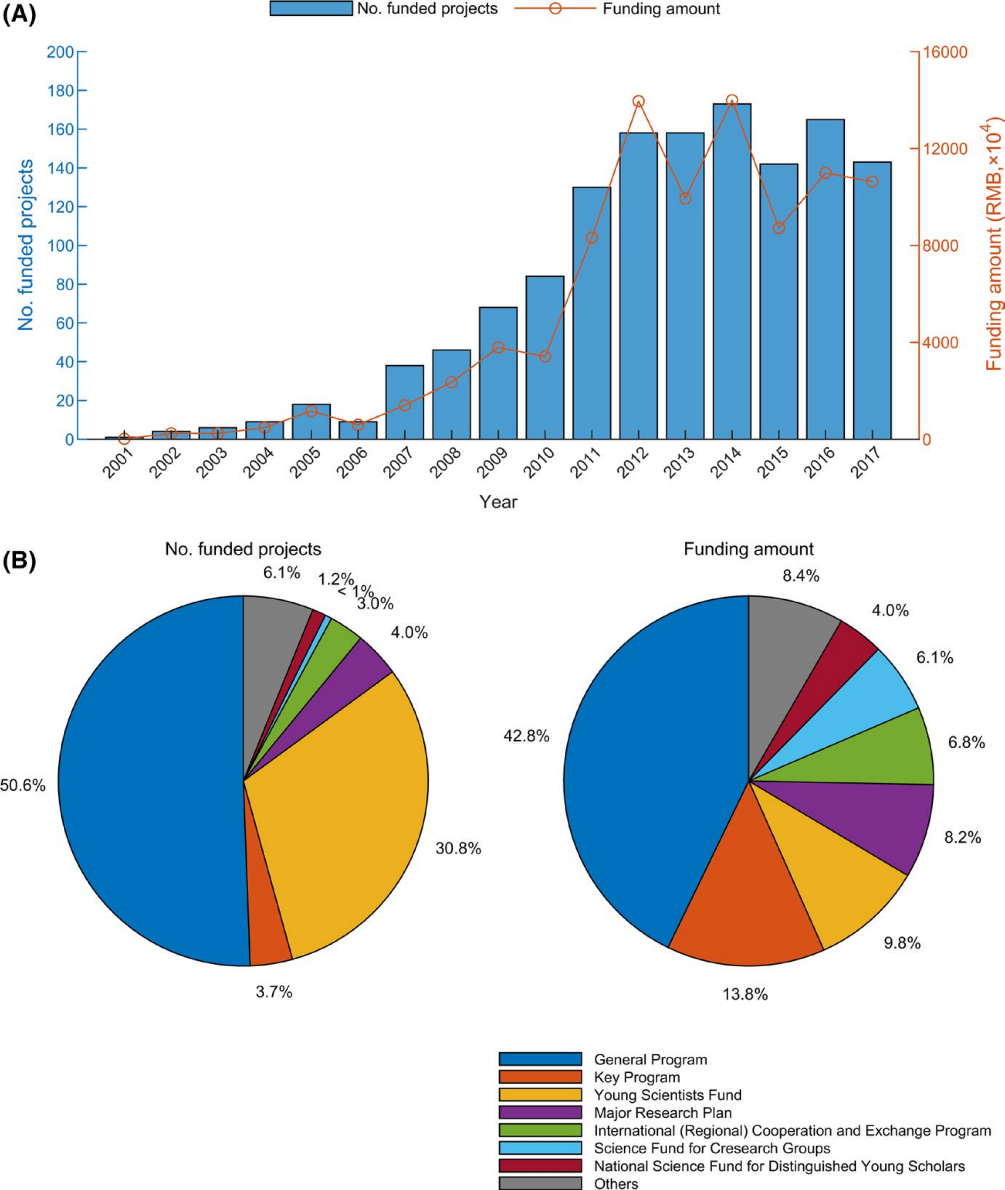
Funding from the NSFC in the fMRI field. (A) The numbers and amounts of fMRI‐related projects founded by the NSFC from 2001 to 2017. (B) The numbers and amounts of different categories of programs in the NSFC

We retrieved the raw dataset of all funded research proposals relating to fMRI from the NSFC official website (https://isisn.nsfc.gov.cn/egrantweb/). Given that the information for projects funded after 2018 was not fully recorded, we restricted our analysis to projects funded from 2001 to 2017. Over this period, a total of 1352 projects and 902.6 million RMB (~US $138.9 million) were funded by the NSFC. Both the number of projects and funding amounts supported by the NSFC have increased remarkably, particularly since 2010 (Figure 1A). In 2001, only one project was supported with 0.18 million RMB (~US$ 0.03 million) of funding. In contrast, in 2014, the number of projects reached 173, and the amount of funding increased to 139.91 million RMB (US $21.5 million). Among the NSFC‐funded projects related to fMRI, 50.6% (684 projects) were under the funding scheme of the General Program (386.4 million RMB, 42.8% in amounts), which is a primary funding type that supports recipient scientists in freely selecting their research themes (Figure 1B). The second largest project type was the Young Scientists funding scheme (30.8%, 416% projects, and 9.8% in amounts, 88.5 million RMB), which is a kind of project allowing young researchers to freely engage in the study of key scientific research. Notably, although the Key Program only accounted for 3.7% (50 projects) of the projects, it provided 13.8% of the funding (125.0 million RMB). This corresponds to its responsibility to support researchers in conducting in‐depth, systematic, and innovative research in the direction of sound research. Finally, the influential talent‐oriented projects that aim to support adept scholars and research teams, including the Excellent Young Scientist Fund, National Science Fund for Distinguished Young Scholars and Science Fund for Creative Research Groups, supported a total of 41 projects (110.9 million RMB) related to fMRI research.

### 1.2 Topic and disciplines of NSFC grants for fMRI research

**FIGURE 2:**
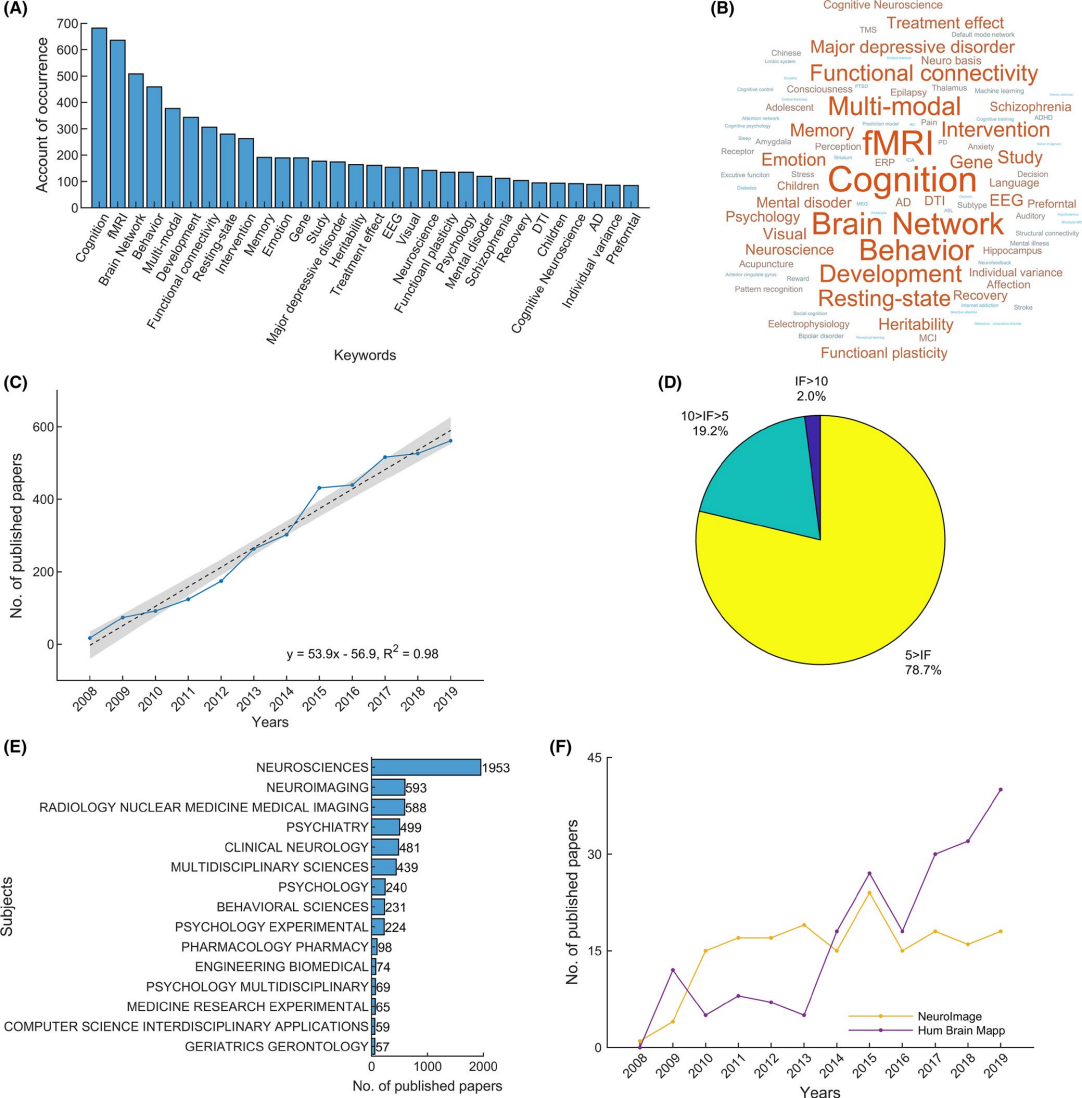
Top keywords and published articles of fMRI research projects funded by the NSFC. (A) The account of occurrence of the top 30 keywords. (B) A word cloud representing the keywords that occurred over 10 times. A larger font size indicates more frequent occurrence. (C) The numbers of published articles that were funded the NSFC that comprised fMRI projects. (D) Percentages of published articles in journals with different impact factors. (E) Discipline categories of the published articles. (F) The number of articles that comprised fMRI projects, were funded by the NSFC and were published in *NeuroImage* and *Human Brain Mapping*

To examine the key topic of the fMRI research funded by NSFC, we conducted a word frequency analysis of the title, abstract, and keyword of the project. The top 15 words with the highest frequency included cognition, brain network, behavior, multimodal, development, functional connectivity, resting‐state, intervention, memory, emotion, gene, study, major depressive disorder, heritability, and treatment effect (Figure 2A,B). These widely distributed topics represent a broad scope of sponsorship by the NSFC funding schemes covering almost all subfields of fMRI research, from the basic fMRI analytical methodology to application research into brain mechanisms for cognition and behavior, normal development, and neuropsychiatric disorders. To better illustrate the NSFC support in specific research areas or topics, including data analysis methods, brain disorders, brain development, and neuroimaging techniques, we further analyzed these fMRI research projects’ title, keyword, and abstract. We found that 73 projects (78.0 million RMB) were related to computation and analysis methods, 932 projects (629.1 million RMB) were supported for brain disorders, and 212 projects (139.7 million RMB) were involved in brain development. However, only eight projects (6.81 million RMB) aimed at developing imaging theory and equipment. With support from the NSFC, fMRI research in China has developed rapidly. We conducted a publication search in the Web of Science Core Collection (http://apps.webofknowledge.com/) using all of the grant numbers of the NSFC projects. A total of 3732 articles were published in peer‐reviewed journals, with a significant linear yearly increase of *β* = 53.9 (articles published in 2020 were not included due to their incomplete records) (Figure 2C). Over 20% of the articles were published in journals with an impact factor (IF) higher than 5, among which 76 articles (accounting for 2.0% of the total number) were published in top journals with an IF higher than 10 (Figure 2D). Regarding the fund categories, the General Program, and the Young Scientists Fund supported the largest number of the published articles, with 2643 and 1472, respectively. Meanwhile, although the Science Fund for Creative Research Groups only supported 186 articles, the percentages of the articles published in journals with an IF higher than 10 (5.9%), or between 5 and 10 (41.4%) were the highest among all fund categories (Table S1). According to the discipline categories defined by the Web of Science, these articles covered a broad scope of disciplines, particularly in neuroscience, neuroimaging, radiology nuclear medicine, psychiatry, clinical neurology, and multidisciplinary sciences (Figure 2E). Notably, in the NeuroImaging subfield, 405 articles in total have been published in the top two journals, *Neuroimage* and *Human Brain Mapping*. Since 2010, 15 to 20 articles have been published on Neuroimage each year in a stable manner. The number of articles published in *Human Brain Mapping* has seen a remarkable increase since 2013, with 40 papers published in 2019 (Figure 2F). This publication record suggests the remarkably increasing amount of fMRI‐related articles in China; however, high‐profile journal papers are still lacking.

### 1.3 The institutes and their cooperation with fMRI research

**FIGURE 3:**
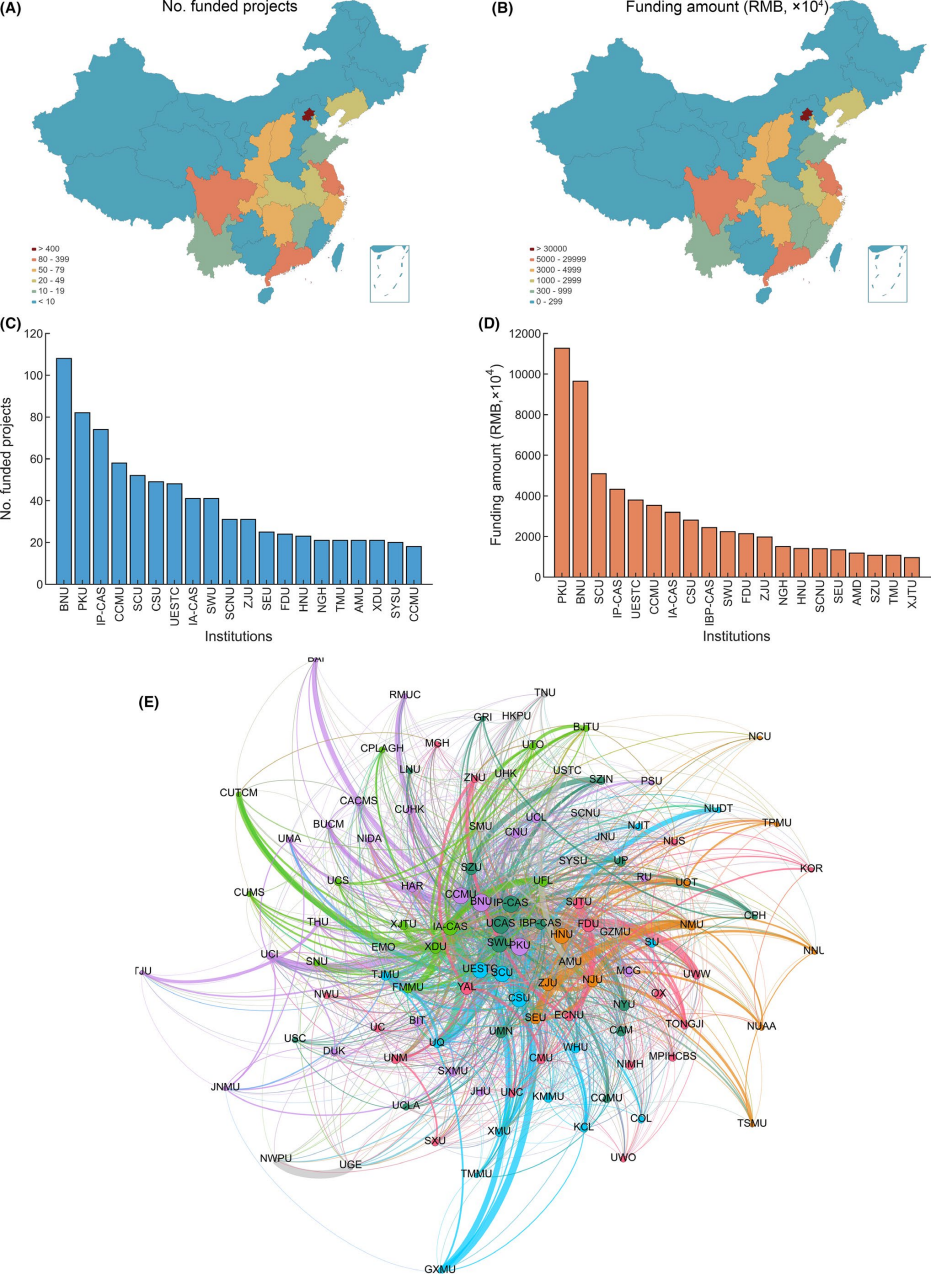
Geographic distribution and institutions of fMRI projects funded by the NSFC. (A) Funded projects and (B) amounts by municipalities and provinces in China. (C) The numbers and (D) amounts of fMRI‐related projects funded by the NSFC and conducted in different institutions. (E) The co‐occurrence network of the institutions. Each node indicates an institution and edge size indicates the amount of co‐occurrence in papers of a given pair of institutions. Nodal size represents degree centrality and nodal color indicates the community assignment. Abbreviation for the top institutions (above the mean plus 1.5 standard deviations in degree centrality): Beijing Normal University, BNU; University of Electronic Science and Technology of China, UESTC; Institute of Psychology of the Chinese Academy of Sciences, IP‐CAS; Institute of Automation of the Chinese Academy of Sciences, IA‐CAS; University of the Chinese Academy of Sciences, UCAS; Peking University, PKU; Capital Medical University, CCMU; Central South University, CSU; Hangzhou Normal University, HNU; Xidian University, XDU; Sichuan University, SCU; and Southwest University, SWU. See Table S5 for all abbreviations

Between 2001 and 2017, 183 registered research institutions were funded by the NSFC in the field of fMRI. Most of the top‐funded institutions are geographically concentrated in metropolitan areas of China. The most economically developed municipalities and provinces of China, including Beijing, Shanghai, Jiangsu, Sichuan, and Guangdong, received 45.4% (884 projects, 70.3% in amounts, 652.2 million RMB) of the total funding awarded for fMRI research by the NSFC, whereas relatively underdeveloped provinces, such as Ningxia, Xinjiang, and Guizhou, received funding for fewer than 10 projects (Figure 3A,3B). Beijing, the capital city of China, has 36 funded research institutions that worked on a total of 464 projects funded with 389.1 million RMB, including 241 General Program (135.2 million RMB), 26 Key Program (62.5 million RMB), 107 Young Scientists Fund (22.8 million RMB), 24 Major Research Plan (34.4 million RMB), and 19 International (Regional) Cooperation and Exchange Program (27.4 million RMB). Remarkably, 7 (45 million RMB) of the total 8 Science Fund for Creative Research Groups and 8 (17.8 million RMB) of the total 16 National Science Fund for Distinguished Young Scholars were supported in Beijing, indicating its leading position in China (Table S2 and S3). Top research universities, such as those designated as part of the nation's “985” and “211” strategic development plans for tertiary education, claimed a high proportion of funded projects. For example, the top 5 universities that received fMRI‐related funds from the NSFC are Beijing Normal University (BNU, 108 projects, 96.5 million RMB), Peking University (PKU, 82 projects, 11.3 million RMB), the Institute of Psychology of the Chinese Academy of Sciences (IP‐CAS, 74 projects, 43.2 million RMB), Capital Medical University (CCMU, 58 projects, 35.4 million RMB), and Sichuan University (SCU, 52 projects, 50.8 million RMB, Sichuan) (Figure 3C,3D). With their strong research infrastructures and ability to compete for centralized funding, these universities and institutes have become the veritable powerhouses of fMRI research in China.

To provide a better understanding of the interaction between these universities and institutes regarding fMRI research, we conducted a graph‐theoretical analysis of the article co‐occurrence network. Briefly, we first constructed a co‐occurrence network from the obtained 3732 fMRI‐related published papers, in which the nodes were defined as the research institutes, and the weights of edges were defined as the number of papers that had coauthors in the two research institutes. To simplify the analysis and focus on the top institutes, we restricted the following analysis within a subnetwork compromising institutions with more than 50 co‐occurrences. We then calculated degree centrality for each node. A higher degree centrality of a given node indicates a higher level of cooperation for that specific institute. Accordingly, several universities and institutions were identified as network hubs (above the mean plus 1.5 standard deviations in degree centrality) that exhibited considerably more cooperation, including BNU (Z = 4.7), the University of Electronic Science and Technology of China (UESTC, Z = 3.3), IP‐CAS (Z = 3.3), the Institute of Automation of the Chinese Academy of Sciences (IA‐CAS, Z = 2.8), the University of the Chinese Academy of Sciences (UCAS, Z = 2.6), PKU (Z = 2.6), CCMU (Z = 2.5), Central South University (CSU, Z = 1.9), Hangzhou Normal University (HNU, Z = 1.9), Xidian University (XDU, Z = 1.9), SCU (Z = 1.7), and Southwest University (SWU, Z = 1.7). By analyzing the disciplines of the publications of these typical institutes, we noticed that besides the common interest in the neuroscience field, these institutes also have some distinct investigation directions. BNU, UESTC, IP‐CAS, IA‐CAS, and PKU are more focused in neuroimaging field; BNU, IP‐CAS, PKU, and SWU are in experimental psychological studies; and UESTC, CSU, and SCU are more engaged in psychiatric and clinical neurological investigations (Table S4). We further applied the Louvain community detection algorithm^3^ to identify cooperation clusters among the institutions. We observed a modularity Q = 0.243 for this cooperation network, suggesting that relatively independent but interrelated cooperative communities existed in the network. Specifically, as illustrated in Figure 3E, the cooperation among institutions exhibited geographic‐ and discipline‐related distributions. For example, BNU, CCMU, and PKU, which are located in Beijing, had strong interactions; SCU and UESTC, which are located in Chengdu and both engaged in psychiatric and clinical neurological investigations, demonstrated a close relationship with the community; and IP‐CAS and SWU, which are more focused on experimental psychology and behavioral science research, were grouped into the same cluster. Notably, there was also a large amount of strong intercommunity cooperation, such as connections between BNU and IP‐CAS, links between CCMU and IA‐CAS, and associations between SWU and UESTC. Together, these results suggest close collaboration among domestic institutions in fMRI research.

### 1.4 Representative achievement of fMRI studies in China

Under the support of the NSFC, scholars from China have obtained many achievements in fMRI research fields that have received increasing international attention, particularly regarding data analysis methods of resting‐state fMRI, brain connectome, and computational platforms in addition to their application in brain disorders. Here, we summarize several representative works. Aiming to characterize the spontaneous activity signal of resting‐state fMRI data, Zang and colleagues from IA‐CAS proposed the amplitude of low‐frequency fluctuation (ALFF), which calculates the time series of a single voxel at a specific frequency.^4^ They found significantly higher ALFF values in the medial frontoparietal cortices compared to the average level of the whole brain, where higher regional cerebral blood flow and local oxygen metabolism have been found in previous studies. This suggests that ALFF values may be closely related to the metabolism of spontaneous brain activity. Then, the research group noticed that some brain areas with obvious physiological noise, such as the ventricles and large blood vessels, also have high ALFF values. To suppress the low‐frequency amplitude of the ventricle and improve the gray matter sensitivity of the measurement, they further proposed the fractional amplitude of low‐frequency fluctuation (fALFF), which is calculated as the ratio of the low‐frequency amplitude to the full‐frequency amplitude of the signal at each voxel.^5^ Additionally, to estimate the synchronization of local signals, Zang et al. proposed a regional homogeneity metric, which calculates Kendall's coefficient of concordance between a given voxel and its neighboring voxels.^6^ These three metrics have been widely recognized by domestic and international fMRI research teams; moreover, they have been widely used in scientific research as they have been cited over 3000 times. For example, researchers from the Shenzhen Institute of Neuroscience used ALFF to study the evolutional and developmental patterns of the left inferior frontal gyrus (IFG) in humans and macaques. They showed that the ALFF of the IFG subregions were significantly increased during evolution and were significantly higher in senior‐aged groups.^7^

In the last decade, research into large‐scale functional connectomes derived from fMRI data has become a hotspot in the field of neuroscience. Several research groups in China have made a series of representative achievements in developing investigational methods and assessing the physical substrates of the functional connectome. For example, the research group from BNU systematically evaluated the effects of several key factors, such as different brain parcellation and connection definitions, on the topological measurements of the functional brain networks derived from resting‐state fMRI data; the results of this evaluation have guided the choice of strategies for functional brain network analysis. The researchers found that the topology of functional networks had significant differences when different atlases were used to define network nodes or different null models were adopted.^8^, ^9^ They also proposed several frameworks to characterize the dynamic and directed functional connectivity of networks, including the variable parameter regression model combined with the Kalman filtering method^10^ and the convergent cross mapping approach.^11^ The researchers further systematically evaluated the test‐retest reliability (TRT) of resting‐state functional brain networks using repeated measured scans. Their results showed that most functional hubs exhibited fair to good TRT reliability using intraclass correlation coefficients and suggested that a 6‐minute scan duration was required to reliably detect these functional hubs.^12^ The TRTs of network metrics were sensitive to the scanning orders and intervals, with fair to excellent long‐term reliability (>6 weeks) but lower short‐term reliability (~20 min).^13^ Finally, the researchers also explored the physiological basis of these brain network properties by elaborating on the relationship between highly connected hub regions of large‐scale functional brain networks and the underlying metabolic consumption patterns of the brain. Using multimodal BOLD fMRI and arterial spin labeling perfusion MRI data, the researchers showed a significant positive correlation between the nodal centralities of whole‐brain functional networks and regional cerebral blood flow, especially in the long‐range functional hubs of brain networks. This relationship appeared to be significantly strengthened with increasing working memory task load.^14^

The research group from UESTC made a series of improvements to the estimation of the directed connections in functional brain networks. They developed a kernel canonical correlation based on multivariate nonlinear Granger causality to explore local directed network connectivity at different scales.^15^, ^16^ Using multivariate Granger causality analysis, they constructed a whole brain‐directed influence brain network and found that the directed functional network followed a small‐world configuration with several medial and lateral frontoparietal regions acting as network hubs; these hubs were either affected by or exerted an influence on other regions.^17^ The confounding effect of a hemodynamic response function (HRF) and the conditioning of a large number of variables in the presence of short time series are two core issues for reconstructing directed functional brain networks using fMRI data. Aiming to overcome these issues, the researchers proposed a blind deconvolution approach to recover effective connectivity of brain networks from resting‐state fMRI data, which considers resting‐state fMRI data as “spontaneous event‐related” to extract a region‐specific HRF and use it in deconvolution.^18^ The researchers further proposed a partially conditioned Granger causality to cope with the redundancy and dimensionality curse in evaluating effective connectivity from fMRI data. The researchers constructed a high‐resolution directed functional brain network at the voxel level and depicted several voxelwise hubs of incoming and outgoing connections, which were mostly located in the default mode network (DMN).^18^, ^19^ Collaborated with researchers from CCMU, the research group from UESTC developed an approach to reliably identify homologous functional regions in each individual and showed that aligning data using the homologous functional regions derived from resting‐state fMRI can significantly improve the study of resting‐state functional connectivity, task fMRI activations, and brain‐behavior associations.^20^

Many of the proposed analysis methods have been embedded in several domestically toolboxes and platforms for neuroimaging processing, network computation, and visualization. Most of these toolboxes are published on neuroscience websites and are freely available to researchers using fMRI. For example, the graph‐theoretical network analysis (GRETNA) toolbox enables the construction and topological analysis of networks based on MATLAB code with parallel acceleration (http://www.nitrc.org/projects/gretna).^21^ Based on statistical parametric mapping (SPM), the REST^22^ and Data Processing Assistant for Resting‐State fMRI (DPARSF) provides a pipeline for resting‐state fMRI data analysis (http://www.nitrc.org/projects/dparsf).^23^ BrainNet Viewer provides flexible functions for the visualization of measurements derived from neuroimages and topological architectures of brain networks (http://www.nitrc.org/projects/bnv/).^24^ These toolboxes have been widely used by more than 1000 laboratories worldwide, and they have been used in over 5000 published articles.

The application of fMRI analysis becomes increasingly important when either studying pathophysiology or exploring neuroimaging biomarkers associated with neuropsychiatric disorders. The research group from West China Hospital of SCU applied fMRI techniques to improve the understanding of psychiatric illnesses and treatment effects. The researchers proposed a new discipline in medical sciences, psychoradiology, which uses radiological technologies to unveil patterns of brain abnormalities in patients with psychiatric disorders.^25^ They conducted neuroimaging studies with a considerable number of participants to identify alterations in brain features in patients with psychiatric disorders underlying the disruption of emotion and behavior. Using resting‐state functional connectivity analysis, they revealed different disrupted functional circuits in patients with non‐refractory and refractory depression. Refractory depression is associated with disrupted functional connectivity mainly in thalamocortical circuits, whereas non‐refractory depression is associated with more distributed decreased connectivity in the limbic‐striatal‐pallidal‐thalamic circuit.^26^ Cooperating with a research group from BNU, the research group from West China Hospital of SCU conducted the first whole‐brain functional network analysis in drug‐native, first‐episode major depressive disorder patients. The patient group showed a shorter path length and a higher global efficiency, implying a shift toward randomization in their brain networks. Furthermore, altered nodal centralities in depressed patients were predominately located in the DMN regions, primary sensory cortex, and caudate nucleus.^27^ The investigators from HNU and IP‐CAS collaborated with 17 sites over China and studied the functional connectivity within the DMN in patients with depression. They showed that the reduction of functional connectivity only in recurrent MDD, but not in first‐episode drug‐naïve MDD. The altered functional connectivity was associated with medication usage but not with disease duration.^28^ In schizophrenia, the research group from West China Hospital of SCU found significant correlations between symptom scores for thought disturbance and temporo‐putamen connectivity and between negative symptoms and temporo‐precuneus connectivity.^29^ Using ALFF values to characterize regional cerebral function, they found that antipsychotic therapy can increase regional spontaneous activity in widespread brain areas, providing further understanding of antipsychotic drugs at a complex system level.^30^ Deep brain stimulation (DBS) is a powerful therapy strategy for movement disorders; however, its neural mechanisms remain unclear. Using simultaneous DBS‐fMRI techniques, Researchers from Peking University revealed robust BOLD responses in the basal ganglia‐thalamocortical network in a frequency‐dependent manner in Parkinsonian rats.^31^ The research group from Tsinghua University investigated the whole‐brain functional effects of subthalamic nucleus stimulation in patients with Parkinson's disease. They showed that a circuit involving the globus pallidus internus, thalamus, and deep cerebellar nuclei was significantly activated, whereas another circuit involving the primary motor cortex, putamen, and cerebellum showed deactivation. These two distinct neurocircuits were related to divergent symptoms, providing a novel understanding of DBS’s neural mechanisms in Parkinson's disease.^32^ Developmental disorders can also be companied with brain alterations in adults. A study of the collaborations between BNU and PKU examined the alterations of cerebral blood flow and resting‐state functional connectivity in adults with attention‐deficit/hyperactivity disorder. They showed significantly decreased cerebral blood flow in the large‐scale resting‐state networks, including the ventral attention, somatomotor, and limbic networks in patients compared with the controls.^33^ Regarding physiological pain, using task fMRI, researchers from Peking University found that the pain‐induced activation in the dorsal anterior cingulate cortex and bilateral insula was significantly reduced after individuals performed altruistic actions, suggesting that incurring personal costs to help others can relieve painful feelings in human performers.^34^

## 2 CONCLUSION

In general, both the number of projects and amount of funding invested in research using fMRI from the NSFC have increased substantially in the 21st century. These projects have promoted the rapid development of fMRI research in China and have fueled several representative achievements in fMRI computation methods and their applications in brain disorders. More importantly, several fMRI research teams with specialized expertise have been developed in China, and a highly cooperative environment has been created. However, domestic research into neuroimaging techniques is still not enough, especially in developing imaging theory and equipment. Most of the MRI scanners reported in the NSFC‐funded projects are imported instruments from abroad. Nevertheless, the domestic manufacturers such as United Imaging have made great progress in MR scanners and could gain a place in fMRI research in future. It is worth noting that the clinical translational application of fMRI research needs to make further efforts. The China Brain Project is about to launch; this project covers basic research on neural mechanisms underlying cognition, brain development, translational research for the diagnosis and intervention of brain diseases, and brain‐inspired intelligence technology. As a technological tool for basic research, fMRI will play an important role in many research topics. It is foreseeable that future research trends of fMRI will rely more on multicenter imaging big data with multidimensional biological variables (eg, imaging, genetic, demographic, cognitive, and clinical measures) and focus more on individual differences and individual uniqueness. A large collaborative project integrating multicenter fMRI datasets from ten domestic research institutes and clinical hospitals has revealed reproducible disruptions in the functional connectome in patients with major depressive disorder,^35^, ^36^ offering a promising paradigm for the understanding of the pathologies and exploration of biomarkers for clinical diagnosis and treatment of psychiatric disorders. Combining brain stimulation techniques and fMRI can offer a powerful tool for elucidating brain functions. For example, modulating the activity of the right temporoparietal junction can lead to changes in the social framing effect but not in nonsocial conditions.^37^ Thus, future studies with simultaneous brain stimulation and neuroimaging could provide causal interpretations for the large‐scale functional brain activity and connectivity observed by fMRI. Moreover, by searching keywords related to machine learning in the papers funded by NSFC fMRI projects, we observed a considerable increase from 2008 to 2019, particularly after 2017 (Figure S1). This increase indicates that future fMRI research will integrate machine learning methods more to reveal the neuronal features that underlie individual behaviors and brain disorders and identify imaging biomarkers for predicting cognitive performance, normal development, disease diagnosis, and prognosis. These are also hot topics for fMRI research that the NSFC needs to pay attention in future.

## CONFLICT OF INTEREST

The authors declare that they have no conflict of interest.

## ETHICAL APPROVAL

This is a meta‐analysis. Our hospital Ethics Committee has confirmed that no ethical approval is required.

## CONSENT FOR PUBLICATION

Publication is approved by all authors and tacitly or explicitly by the responsible authorities where the work was carried out.

## Supporting information

Supplementary Material

Fig S1

## Data Availability

The data that support the findings of this study are available on request from the corresponding author. The data are not publicly available due to privacy or ethical restrictions
