# Supplementary material for: Functional magnetic resonance imaging research in China
Source: CNS Neurosci Ther. 2021 Sep 7;27(11):1259–67. doi: 10.1111/cns.13725 (PMC8504522; doi:10.1111/cns.13725)
Supplement: Supplementary file 1 — Supplementary Material [file CNS-27-1259-s001.docx]

**Supplementary Information**

**Table S1. Published articles supported by different fund categories.**

| **Fund Category** | **Published paper, n** | **Published paper in journals with an IF between 5 and 10, n (%)** | **Published papers in journals with an IF larger than 10, n (%)** |
| --- | --- | --- | --- |
| General Program | 2643 | 506 (19.1%) | 42 (1.6%) |
| Key Program | 812 | 216 (26.6%) | 21 (2.6%) |
| Young Scientists Fund | 1472 | 260 (17.7%) | 20 (1.4%) |
| Major Research Plan | 466 | 141 (30.3%) | 18 (3.9%) |
| International (Regional) Cooperation and Exchange Program | 410 | 125 (30.5%) | 13 (3,2%) |
| Science Fund for Creative Research Groups | 186 | 77 (41.4%) | 11 (5.9%) |
| National Science Fund for Distinguished Young Scholars | 216 | 59 (27.3%) | 7 (3.2%) |
| Others | 414 | 83 (20.1%) | 9 (2.2%) |

**Table S2. Number of different fund categories in different provinces.**

| Province | General Program | Key Program | Young Scientists Fund | Major Research Plan | International (Regional) Cooperation and Exchange Program | Science Fund for Creative Research Groups | National Science Fund for Distinguished Young Scholars | Others |
| --- | --- | --- | --- | --- | --- | --- | --- | --- |
| Shanghai | 50 | 1 | 30 | 4 | 4 | 0 | 0 | 0 |
| Yunnan | 2 | 0 | 3 | 0 | 0 | 0 | 0 | 6 |
| Neimenggu | 2 | 0 | 2 | 0 | 0 | 0 | 0 | 0 |
| Beijing | 241 | 26 | 107 | 24 | 19 | 7 | 8 | 32 |
| Jilin | 2 | 0 | 1 | 0 | 0 | 0 | 0 | 0 |
| Sichuan | 49 | 10 | 44 | 7 | 7 | 1 | 3 | 3 |
| Tianjin | 9 | 2 | 19 | 1 | 1 | 0 | 1 | 0 |
| Ningxia | 0 | 0 | 0 | 0 | 0 | 0 | 0 | 2 |
| Anhui | 21 | 1 | 13 | 3 | 0 | 0 | 0 | 2 |
| Shandong | 9 | 0 | 5 | 0 | 0 | 0 | 0 | 0 |
| Shanxi | 5 | 0 | 2 | 0 | 0 | 0 | 0 | 0 |
| Guangdong | 67 | 2 | 40 | 4 | 1 | 0 | 1 | 2 |
| Guangxi | 0 | 0 | 1 | 0 | 0 | 0 | 0 | 5 |
| Xinjiang | 0 | 0 | 0 | 0 | 0 | 0 | 0 | 1 |
| Jiangsu | 42 | 1 | 35 | 3 | 3 | 0 | 1 | 5 |
| Jiangxi | 1 | 0 | 1 | 0 | 0 | 0 | 0 | 11 |
| Hebei | 1 | 0 | 0 | 0 | 0 | 0 | 0 | 0 |
| Henan | 3 | 0 | 3 | 0 | 0 | 0 | 0 | 0 |
| Zhejiang | 28 | 0 | 25 | 5 | 3 | 0 | 0 | 0 |
| Hainan | 0 | 0 | 0 | 0 | 0 | 0 | 0 | 5 |
| Hubei | 10 | 0 | 13 | 1 | 0 | 0 | 0 | 0 |
| Hunan | 38 | 3 | 18 | 1 | 2 | 0 | 1 | 1 |
| Gansu | 0 | 0 | 1 | 0 | 0 | 0 | 0 | 3 |
| Fujian | 5 | 0 | 1 | 0 | 0 | 0 | 0 | 0 |
| Guizhou | 0 | 0 | 0 | 0 | 0 | 0 | 0 | 1 |
| Liaoning | 14 | 0 | 10 | 0 | 0 | 0 | 1 | 1 |
| Chongqing | 44 | 1 | 21 | 0 | 0 | 0 | 0 | 2 |
| Shanxi | 37 | 3 | 20 | 1 | 1 | 0 | 0 | 1 |
| Hongkong | 2 | 0 | 0 | 0 | 0 | 0 | 0 | 0 |
| Heilongjiang | 2 | 0 | 1 | 0 | 0 | 0 | 0 | 0 |

**Table S3. Amounts of different fund categories in different provinces (RMB ×10^4^).**

| Province | General Program | Key Program | Young Scientists Fund | Major Research Plan | International (Regional) Cooperation and Exchange Program | Science Fund for Creative Research Groups | National Science Fund for Distinguished Young Scholars | Others |
| --- | --- | --- | --- | --- | --- | --- | --- | --- |
| Shanghai | 2842.3 | 320 | 641 | 720 | 727.5 | 0 | 0 | 0 |
| Yunnan | 85 | 0 | 65 | 0 | 0 | 0 | 0 | 241 |
| Neimenggu | 122 | 0 | 46 | 0 | 0 | 0 | 0 | 0 |
| Beijing | 13523 | 6248 | 2277.5 | 3440 | 2735 | 4500 | 1780 | 4409.3 |
| Jilin | 124 | 0 | 25 | 0 | 0 | 0 | 0 | 0 |
| Sichuan | 2562 | 2352 | 936.5 | 750 | 1210.5 | 1050 | 600 | 520 |
| Tianjin | 432 | 575 | 395 | 70 | 45 | 0 | 400 | 0 |
| Ningxia | 0 | 0 | 0 | 0 | 0 | 0 | 0 | 100 |
| Anhui | 1119 | 315 | 278 | 550 | 0 | 0 | 0 | 60 |
| Shandong | 521 | 0 | 114 | 0 | 0 | 0 | 0 | 0 |
| Shanxi | 310 | 0 | 50 | 0 | 0 | 0 | 0 | 0 |
| Guangdong | 3904 | 555 | 840 | 455 | 80 | 0 | 200 | 26 |
| Guangxi | 0 | 0 | 20 | 0 | 0 | 0 | 0 | 217 |
| Xinjiang | 0 | 0 | 0 | 0 | 0 | 0 | 0 | 49 |
| Jiangsu | 2303 | 280 | 722.5 | 220 | 510 | 0 | 200 | 783 |
| Jiangxi | 55 | 0 | 23 | 0 | 0 | 0 | 0 | 439 |
| Hebei | 70 | 0 | 0 | 0 | 0 | 0 | 0 | 0 |
| Henan | 145 | 0 | 55 | 0 | 0 | 0 | 0 | 0 |
| Zhejiang | 1710 | 0 | 528.5 | 960 | 431 | 0 | 0 | 0 |
| Hainan | 0 | 0 | 0 | 0 | 0 | 0 | 0 | 235 |
| Hubei | 573 | 0 | 285 | 100 | 0 | 0 | 0 | 0 |
| Hunan | 2108 | 710 | 374.5 | 70 | 369.2 | 0 | 100 | 130 |
| Gansu | 0 | 0 | 22 | 0 | 0 | 0 | 0 | 117 |
| Fujian | 342 | 0 | 23 | 0 | 0 | 0 | 0 | 0 |
| Guizhou | 0 | 0 | 0 | 0 | 0 | 0 | 0 | 50 |
| Liaoning | 751 | 0 | 216 | 0 | 0 | 0 | 350 | 10 |
| Chongqing | 2512 | 290 | 452 | 0 | 0 | 0 | 0 | 25 |
| Shanxi | 2250 | 855 | 439 | 80 | 20 | 0 | 0 | 130 |
| Hongkong | 150 | 0 | 0 | 0 | 0 | 0 | 0 | 0 |
| Heilongjiang | 126 | 0 | 23 | 0 | 0 | 0 | 0 | 0 |

**Table S4.** **Percentage of published articles in different disciplines of the typical institutes (%).**

| Disciplines | BNU | UESTC | IP-CAS | IA-CAS | PKU | CCMU | CSU | HNU | XDU | SCU | SWU |
| --- | --- | --- | --- | --- | --- | --- | --- | --- | --- | --- | --- |
| Neurosciences | **34.9** | **28.8** | **31.8** | **33.0** | **34.1** | **31.0** | **17.8** | **31.6** | **27.5** | **23.8** | **29.9** |
| Neuroimaging | **11.2** | **10.6** | **10.8** | **10.2** | **11.7** | **6.5** | **7.5** | **9.4** | **9.8** | **9.3** | **8.0** |
| Radiology, Nuclear Medicine & Medical Imaging | **11.0** | **9.9** | **8.6** | **13.3** | **12.1** | **9.5** | 4.2 | **9.8** | **10.8** | **9.5** | **6.2** |
| Multidisciplinary Sciences | **6.7** | **6.2** | **7.9** | **8.3** | **6.8** | **8.4** | **6.4** | **13.8** | **7.1** | **6.8** | **9.0** |
| Psychology, Experimental | **6.0** | 1.4 | **5.5** | 1.9 | **6.5** | 2.2 | 0.0 | 2.0 | 2.4 | 0.7 | **8.0** |
| Psychology | 3.7 | 2.6 | 3.9 | 0.9 | **5.2** | 4.1 | 2.5 | **5.4** | 4.4 | 2.2 | **7.3** |
| Behavioral Sciences | 3.7 | 2.1 | **5.7** | 3.1 | 1.8 | 2.2 | 1.7 | 0.3 | 2.4 | 1.5 | **8.4** |
| Psychiatry | 3.6 | **9.8** | **5.3** | 4.6 | **5.2** | 4.9 | **25.9** | **5.1** | 3.7 | **13.9** | 3.6 |
| Clinical Neurology | 3.0 | **10.2** | 3.1 | 4.9 | 3.2 | **9.3** | **12.8** | **5.7** | **6.4** | **14.3** | 3.6 |
| Integrative & Complementary Medicine | 0.2 | 0.0 | 0.4 | 1.5 | 0.0 | 1.5 | 0.0 | 0.0 | **5.1** | 1.1 | 0.0 |
| Others | 16.1 | 18.5 | 17.1 | 18.2 | 13.3 | 20.3 | 21.2 | 16.8 | 20.3 | 16.8 | 16.1 |

Bold font indicates a percentage of specify discipline above 5%.

BNU, Beijing Normal University; UESTC, University of Electronic Science and Technology of China; IP-CAS, Institute of Psychology of the Chinese Academy of Sciences; IA-CAS, Institute of Automation of the Chinese Academy of Sciences; PKU, Peking University; CCMU, Capital Medical University; CSU, Central South University; HZU, Hangzhou Normal University; XDU, Xidian University; SCU, Sichuan University; and SWU, Southwest University.

**Table S5. Abbreviations for institutes in Figure 5 (sorted in alphabetic order).**

| **Abbreviation** | **Full name** | **Abbreviation** | **Full name** |
| --- | --- | --- | --- |
| **AMU** | Anhui Medical University | **OX** | University of Oxford |
| **BAI** | Banner Alzheimer’s Institute | **PKU** | Peking University |
| **BIT** | Beijing Institute Technology | **PSU** | Pennsylvania State University |
| **BJTU** | Beijing Jiaotong University | **RMUC** | Renmin University of China |
| **BNU** | Beijing Normal University | **RU** | Radboud University Nijmegen |
| **BUCM** | Beijing University of Chinese Medicine | **SCNU** | South China Normal University |
| **CACMS** | China Academy of Chinese Medical Sciences | **SCU** | Sichuan University |
| **CAM** | University of Cambridge | **SEU** | Southeast University |
| **CCMU** | Capital Medical University | **SJTU** | Shanghai Jiao Tong University |
| **CMU** | China Medical University | **SMU** | Southern Medical University |
| **CNU** | Capital Normal University | **SNU** | Shaanxi Normal University |
| **COL** | Columbia University | **SU** | Soochow University |
| **CPH** | Castle Peak Hospital | **SWU** | Southwest University |
| **CPLAGH** | Chinese People's Liberation Army General Hospital | **SXMU** | Shanxi Medical University |
| **CQMU** | Chongqing Medical University | **SXU** | Shanxi University |
| **CSU** | Central South University | **SYSU** | Sun Yat-Sen University |
| **CUHK** | Chinese University of Hong Kong | **SZIN** | Shenzhen Institute of Neuroscience |
| **CUMS** | Capital University of Medical Science | **SZU** | Shenzhen University |
| **CUTCM** | Chengdu University of Traditional Chinese Medicine | **THU** | Tsinghua University |
| **DUK** | Duke University | **TJMU** | Tianjin Medical University |
| **ECNU** | East China Normal University | **TJU** | Tianjin University |
| **EMO** | Emory University | **TMMU** | Third Military Medical University |
| **FDU** | Fudan University | **TNU** | Tianjin Normal University |
| **FMMU** | Fourth Military Medical University | **TONGJI** | Tongji University |
| **GRI** | Griffith University | **TPMU** | Taipei Medical University |
| **GXMU** | Guangxi Medical University | **TSMU** | Taishan Medical University |
| **GZMU** | Guangzhou Medical University | **UC** | University of Chicago |
| **HAR** | Harvard University | **UCAS** | University of the Chinese Academy of Science |
| **HKPU** | Hong Kong Polytechnic University | **UCI** | University of California, Irvine |
| **HNU** | Hangzhou Normal University | **UCL** | University College London |
| **IA-CAS** | Institute of Automation, Chinese Academy of Science | **UCLA** | University of California, Los Angeles |
| **IBP-CAS** | Institute of Biophysics, Chinese Academy of Science | **UCS** | University of California, San Diego |
| **IP-CAS** | Institute of Psychology, Chinese Academy of Science | **UESTC** | University of Electronic Science and Technology of China |
| **JHU** | Johns Hopkins University | **UFL** | University of Florida |
| **JNMU** | Jining Medical University | **UGE** | University of Georgia |
| **JNU** | Jinan University | **UHK** | University of Hong Kong |
| **KCL** | King’s College London | **UMA** | University of Macau |
| **KMMU** | Kunming Medical University | **UMN** | University of Minnesota |
| **KOR** | Korea University | **UNC** | University of North Carolina at Chapel Hill |
| **LNU** | Liaoning Normal University | **UNM** | University of New Mexico |
| **MCG** | McGill University | **UOT** | University of Ottawa |
| **MGH** | Massachusetts General Hospital | **UP** | University of Pennsylvania |
| **MPIHCBS** | Max Planck Institute for Human Cognitive and Brain Sciences | **UQ** | University of Queensland |
| **NCU** | Nanchang University | **USC** | University of Southern California |
| **NIDA** | National Institute on Drug Abuse | **USTC** | University of Science and Technology of China |
| **NIMH** | National Institute of Mental Health | **UTO** | University of Toronto |
| **NJIT** | New Jersey Institute of Technology | **UWO** | University of Western Ontario |
| **NJU** | Nanjing University | **UWW** | University of Warwick |
| **NMU** | Nanjing Medical University | **WHU** | Wuhan University |
| **NNU** | Nanjing Normal University | **XDU** | Xidian University |
| **NUAA** | Nanjing University of Aeronautics and Astronautics | **XJTU** | Xi’an Jiaotong University |
| **NUDT** | National University of Defense Technology | **XMU** | Xinxiang Medical University |
| **NUS** | National University of Singapore | **YAL** | Yale University |
| **NWPU** | Northwestern Polytechnical University | **ZJU** | Zhejiang University |
| **NWU** | Northwestern University | **ZNU** | Zhejiang Normal University |
| **NYU** | New York University |  |  |

**
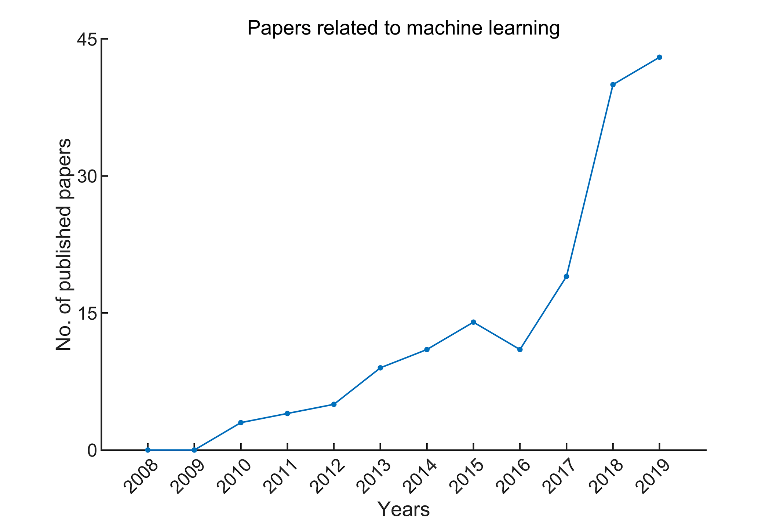
**

**Figure S1. The numbers of machine learnings-related articles that were funded the NSFC that comprised fMRI projects.** The machine learning-related papers were identified that they have any of the keywords “machine learning”, “deep learning”, “support vector”, and “lasso” in title, keyword, or abstract.
