# Supplementary figures and images for: Functional magnetic resonance imaging research in China
Source: CNS Neurosci Ther. 2021 Sep 7;27(11):1259–67. doi: 10.1111/cns.13725 (PMC8504522; doi:10.1111/cns.13725)

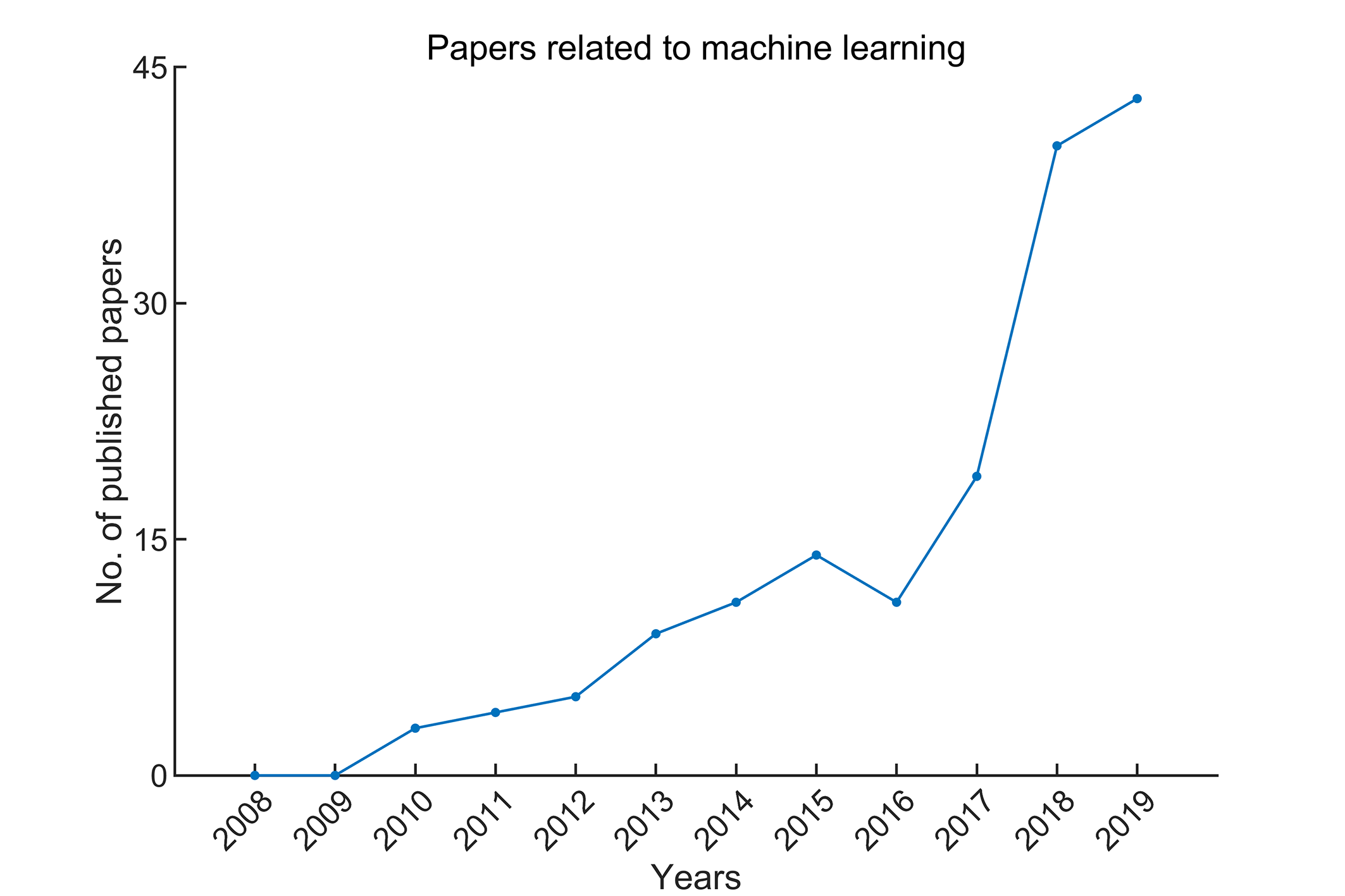

Supplement: Supplementary file 2 — Fig S1 [file CNS-27-1259-s002.tif]
